# Case report: Plasmablastic neoplasm with multinucleated giant cells—Analysis of stemness of the neoplastic multinucleated giant cells

**DOI:** 10.3389/fonc.2022.1023785

**Published:** 2022-11-29

**Authors:** Narumi Otsuka-Kamakura, Yoshiya Sugiura, Toshiki Yamazaki, Naomi Shimizu, Nobuyuki Hiruta

**Affiliations:** ^1^ Department of Pathology, Sakura Hospital, Toho University Medical Center, Sakura, Japan; ^2^ Department of Surgical Pathology, Sakura Hospital, Toho University Medical Center, Sakura, Japan; ^3^ Division of Pathology, The Cancer Institute, Japanese Foundation for Cancer Research, Tokyo, Japan; ^4^ Department of Hematology, Sakura Hospital, Toho University Medical Center, Sakura, Japan

**Keywords:** plasmablastic neoplasm, multinucleated giant cell, cancer stem cell, Yamanaka factors, Mib1/Ki67 index

## Abstract

Cancer stem cells have the capability of self-renewal and multipotency and are, therefore, associated with tumor heterogeneity, resistance to chemoradiation therapy, and metastasis. The hypothesis that multinucleated giant cells, which often emerge following chemo- and/or radiotherapy, serve as cancer stem cells has not been fully evaluated. Although a previous study demonstrated that these cells functioned as stem cells, only low levels of Yamanaka factors were expressed, contrasting with the high expression seen from their gestated first-generation mononuclear cells. Herein, we report a case of a plasmablastic neoplasm with multinucleated giant cells that were analyzed for stemness to test the above hypothesis. The patient was a male in his 80s who had a plasmablastic neoplasm that was not easily distinguishable as plasmablastic lymphoma versus plasma cell myeloma of plasmablastic type. Lymph node biopsy showed predominant mononuclear cell proliferation with admixed multinucleated giant cells. Immunohistochemistry and *in situ* hybridization showed that both multinucleated and mononuclear cells had the same profile: CD138(+), light chain restriction of κ>λ, cyclin D1(+), CD68(-), EBER-ISH (+). These results suggested that both cell types were neoplastic. In accordance with the previous study, the multinucleated giant cells showed low expression of Yamanaka factors, which were highly expressed in some of the mononuclear cells. Furthermore, the multinucleated giant cells showed a much lower proliferative activity (Mib1/Ki67 index) than the mononuclear cells. Based on these results, the multinucleated giant cells were compatible with cancer stem cells. This case is expected to expand the knowledge base regarding biology of cancer stem cells.

## Introduction

Plasmablastic lymphoma (PBL) is an aggressive B-cell lymphoma with plasmablastic features that occurs in immunodeficient patients and is usually associated with Epstein-Barr virus (EBV) infection. It was first reported as lymphoma of the oral cavity in a human immunodeficiency virus (HIV)-infected patient (1). However, many cases have since then been reported that involve different localizations while also occurring in patients who are HIV-negative (2).

Plasma cell myeloma (PCM) is a plasma cell neoplasm that commonly produces monoclonal immunoglobulin (M-protein). Some cases of extraosseous PCM showing severe atypia are classified as plasmablastic PCM (PPCM). It is often difficult to differentiate between PBL and PPCM (**Supplemental Table 1**) (3); thus, in such cases, a diagnosis of plasmablastic neoplasm (PBN) is made (4). There have been several reports of cases of PBL or PBN containing neoplastic multinucleated giant cells (5–7).

Recent *in vitro* and *in vivo* studies mainly conducted in cases of ovarian cancer have proposed the hypothesis that multinucleated giant cells serve as cancer stem cells and are associated with resistance to chemotherapy and the potential for metastasis (8, 9). To the best of our knowledge, this hypothesis has not been tested using surgical pathological analysis.

Herein, we present a case of PBN predominantly consisting of mononuclear cells with admixed multinucleated giant cells that were analyzed for stemness to test the above hypothesis.

## Case description

**Figure 1:**
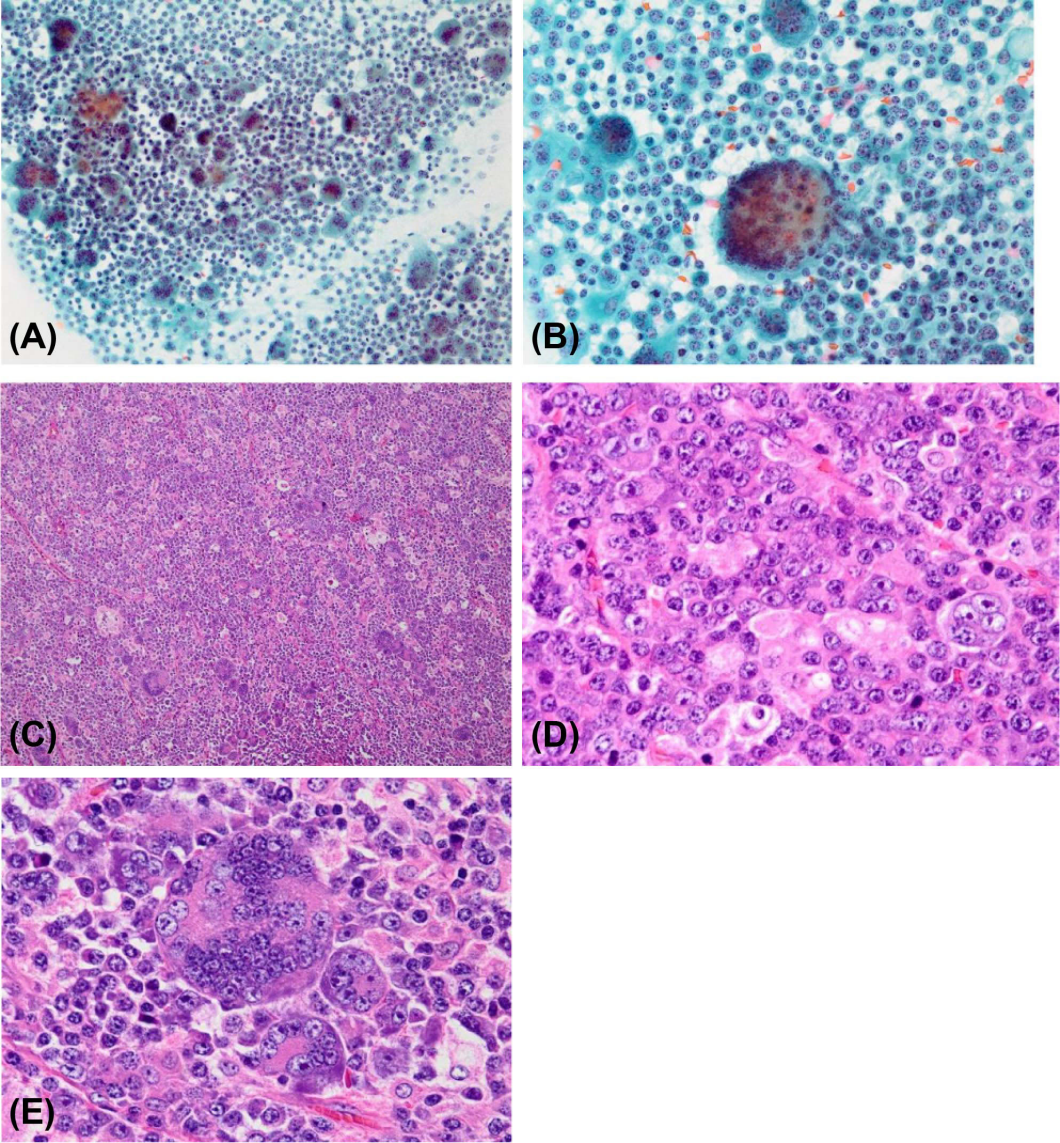
Cytological findings. **(A)** Proliferation of mononuclear cells admixed with multinucleated giant cells (Papanicolaou staining, original magnification ×20); **(B)** Multinucleated giant cells with dozens of nuclei (Papanicolaou staining, original magnification ×40). Histological findings. **(C)** The lymph node was almost replaced by the tumor, and its original architecture was lost (hematoxylin and eosin staining; original magnification ×10); **(D)** Predominant mononuclear neoplastic cells with prominent nucleoli and basophilic cytoplasm (hematoxylin and eosin staining; original magnification ×60); **(E)** Multinucleated giant cells with dozens of nuclei (hematoxylin and eosin staining; original magnification ×60).

A man in his 80s presented to our hospital with a chief complaint of a cervical mass. He had a history of angina and idiopathic interstitial pneumonia but had no overt immune deficiency. Physical examination showed enlarged lymph nodes on the right side of the neck. Computed tomography revealed enlarged cervical lymph nodes and involvement of the mandible and Th1 vertebral body (**Supplemental Figure 1**). Blood analysis showed increased serum immunoglobulin G (IgG) levels (**Supplemental Table 2**), and serum immunofixation electrophoresis detected IgG-κ type M-protein (**Supplemental Figure 2**) which demonstrates the results of serum immunofixation electrophoresis). A fine-needle aspiration biopsy of a cervical lymph node was performed, and the cytological findings suggested plasma cell neoplasm (**Figure 1**). Excisional lymph node biopsy was also performed, and the chromosome analysis revealed a deletion in chromosome 1 and two marker chromosomes (**Supplemental Table 2**), while chromosome 8 was not involved. The final pathological diagnosis was PBN, as described below. The tumor was chemotherapy-resistant, and the patient died 4 months after diagnosis.

### Cytological and histological analysis

For cytological analysis, the cervical lymph node specimen obtained using fine needle aspiration was sprayed on glass slides, and the excisional biopsy specimen was sliced and placed on glass slides. These glass slides were quickly fixed with ethanol for Papanicolaou staining or air-dried and fixed with methanol for Giemsa staining. For histological analysis, tissues were processed following standard procedures. Formalin-fixed paraffin-embedded blocks were cut into 4-μm-thick sections and stained with hematoxylin and eosin.

The analysis showed diffusely proliferating mononuclear cells admixed with multinucleated giant cells. Because of the marked tumor invasion, the original architecture of the lymph node was almost lost. The mononuclear cells had eccentric round nuclei and a basophilic cytoplasm, exhibiting features of plasmablastic or plasma cells. The nuclei had prominent nucleoli and manifested anisokaryosis and irregular chromatin distribution. The multinucleated giant cells had dozens of nuclei, mimicking osteoclasts (**Figure 1**).

### Immunohistochemistry and EBV detection

For immunohistochemistry, unstained specimens were submerged in either a sodium citrate buffer at 97˚C for 20 min or Tris-EDTA buffer at 95°C for 45 minutes or incubated with proteinase K at 37°C for 30 min to retrieve epitopes. Immunostaining was performed using Envision TM FLEX Target Retrieval Solution, High pH (50×) (Agilent, Santa Clara, CA, USA). Specimens underwent immunostaining for CD138, multiple myeloma oncogene-1, CD20, CD79a, CD68, Epstein-Barr nuclear antigen 2 (EBNA2), Cyclin D1, IgG, light chain restriction of κ and λ, cytokeratin AE1/AE3, octamer-binding transcription factor 4 (OCT4), Krüppel-like factor 4 (KLF4), c-Myc, SRY (sex-determining region Y)-box 2 (SOX2), and Mib1/Ki67 (**Supplemental Table 3** which shows the primary antibodies used).

EBV RNA was detected using EBV-encoded RNA *in-situ* hybridization (EBER-ISH). The paraffin-embedded sections (thickness, 4 µm) were dewaxed with xylene, treated with proteinase K, and hybridized with fluorescein isothiocyanate-labeled EBER peptide nucleic acid probe (Agilent). After incubation with anti-fluorescein isothiocyanate-conjugated rabbit polyclonal antibody and polymer horseradish peroxidase-labeled anti-rabbit IgG antibody, slides were covered with diaminobenzidine + chromogen (Agilent).

**Figure 2:**
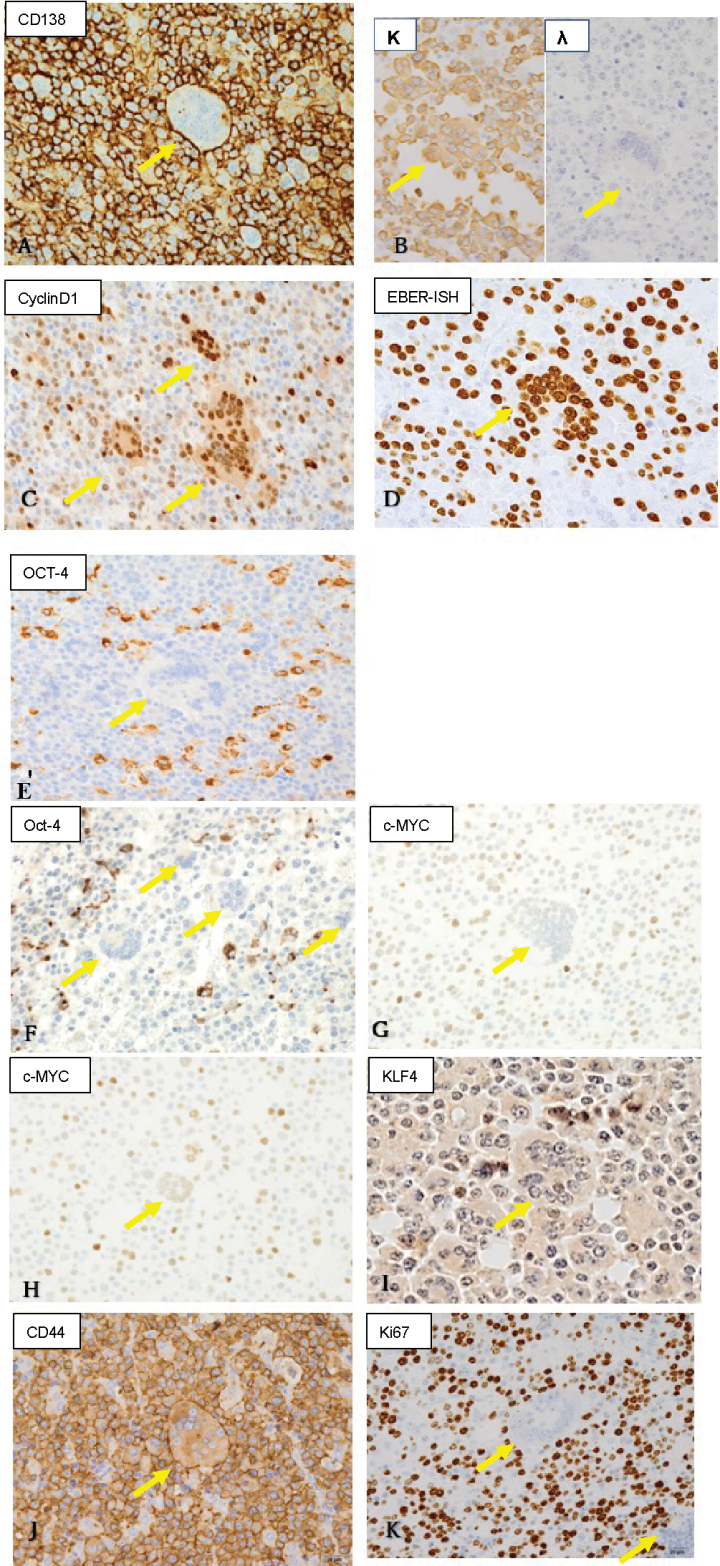
Immunohistochemistry and Epstein-Barr virus-encoded RNA (EBER) in-situ hybridization findings (original magnification ×40). Yellow arrows indicate multinucleated cells. Both multinucleated giant cells and mononuclear cells were positive for **(A)** CD138 (a plasma cell marker; membranous positive), **(B)** light chain restriction of κ>λ (suggesting monoclonality), **(C)** cyclin D1 (a marker for plasma cell myeloma), and **(D)** EBER (suggesting Epstein-Barr virus-associated neoplasm). **(E)** Both cell types were negative for CD68 (a histiocyte marker). **(F)** Mononuclear cells were positive, but multinuclear giant cells were negative for OCT4 (Yamanaka factor). **(G, H)** c-Myc (Yamanaka factor) expression was stronger in mononuclear than in multinucleated cells, and multinucleated cells with a larger number of nuclei **(G)** showed weaker positivity than those with a smaller number of nuclei **(H)**. **(I)** KLF4 (Yamanaka factor) was positive in some of the mononuclear cells, but negative in the multinucleated giant cells. **(J)** CD44 (a marker for cancer stem cells) was positive in both cell types. **(K)** Mib1/Ki67 showed strong positivity in the mononuclear cells, while the multinucleated cells were almost negative.

The results are shown in **Table 1**. First, we assessed the line of differentiation (**Table 1A**). Both the mononuclear and multinucleated cells were positive for CD138 (**Figure 2A**) and revealed light chain restriction of κ>λ (**Figure 2B**), suggesting monoclonal plasma cell proliferation. Furthermore, both cell types were positive for cyclin D1 (**Figure 2C**) and EBER-ISH (**Figure 2D**). However, both cell types were negative for CD68, a marker for histiocytes (**Figure 2E**). These results suggested that not only the mononuclear cells but also the multinucleated giant cells were neoplastic. The multinucleated giant cells were not considered osteoclasts because they expressed features of plasma cell neoplasms while lacking histiocytic features. The neoplastic cells were positive for EBER-ISH, suggesting PBL, but were also positive for cyclin D1, which suggested PPCM. In addition, they did not express EBNA2, a finding that did not support immune suppression and PBL. Due to this inconsistency, we could not differentiate between PBL and PPCM.

**Table 1:** Immunohistochemistry and *in situ* hybridization results.

A. Markers for diagnosis			
Positive	Weakly positive	Negative	
**CD138**	CD3	ALK	CD7
**CD38**	CD56	CD10	CD79a
**c-Myc**	TIA-1	CD2	CD8
**CyclinD1**		CD20	Cytokeratin (AE1/AE3)
**EBER-ISH**		CD30	EMA
**MUM-1**		CD4	Granzyme B
**κ>>λ**		CD5	PAX-5
		CD68 (KP-1)	SOX2
		CD68 (PGM-1)	S-100
			TdT
**B**. **Markers for stemness and proliferative activity**			
**Markers**	**Mononuclear cells**		**Multinucleated giant cells**
**OCT4**	Negative		Positive (strong and partial)
**c-MYC**	Positive (weak and partial)		Positive (strong and partial)
**KLF4**	Negative		Positive (strong and partial)
**SOX2**	Negative		Negative
**CD44**	Positive		Positive
**Mib1/Ki67**	Very low		Very high

These results were common for multinucleated giant cells and mononuclear cells.

EBER-ISH, Epstein-Barr virus-encoded RNA *in-situ* hybridization; MUM-1, multiple myeloma oncogene-1; ALK, anaplastic lymphoma kinase; EMA, epithelial membrane antigen; PAX-5, paired box 5; SOX2, SRY-box transcription factor 2; TdT, terminal deoxynucleotidyl transferase.

Oct4, octamer-binding transcription factor 4; KLF4, Krüppel-like factor 4; SOX2, SRY-box transcription factor 2.

Next, we evaluated the stemness of the mononuclear and multinucleated cells (**Table 1B**). Some of the mononuclear cells exhibited strong cytoplasmic immunopositivity to OCT4, which was not the case with any of the multinucleated cells (**Figure 2F**). As for c-Myc, the mononuclear cells showed stronger nuclear immunopositivity than the multinucleated cells (**Figures 2G, H**). Among the multinucleated cells, those with a larger number of nuclei (**Figure 2G**) showed weaker positivity than those with a smaller number of nuclei (**Figure 2H**). As for KLF4, some of the mononuclear cells showed nuclear positivity, while multinucleated cells were consistently negative (**Figure 2I**). Both mononuclear and multinucleated cells were strongly positive for CD44 (**Figure 2J**) and negative for SOX2. Furthermore, the mononuclear cells had a high Mib1/Ki67 index, while multinucleated cells were scarcely positive (**Figure 2K**).

**Figure 3:**
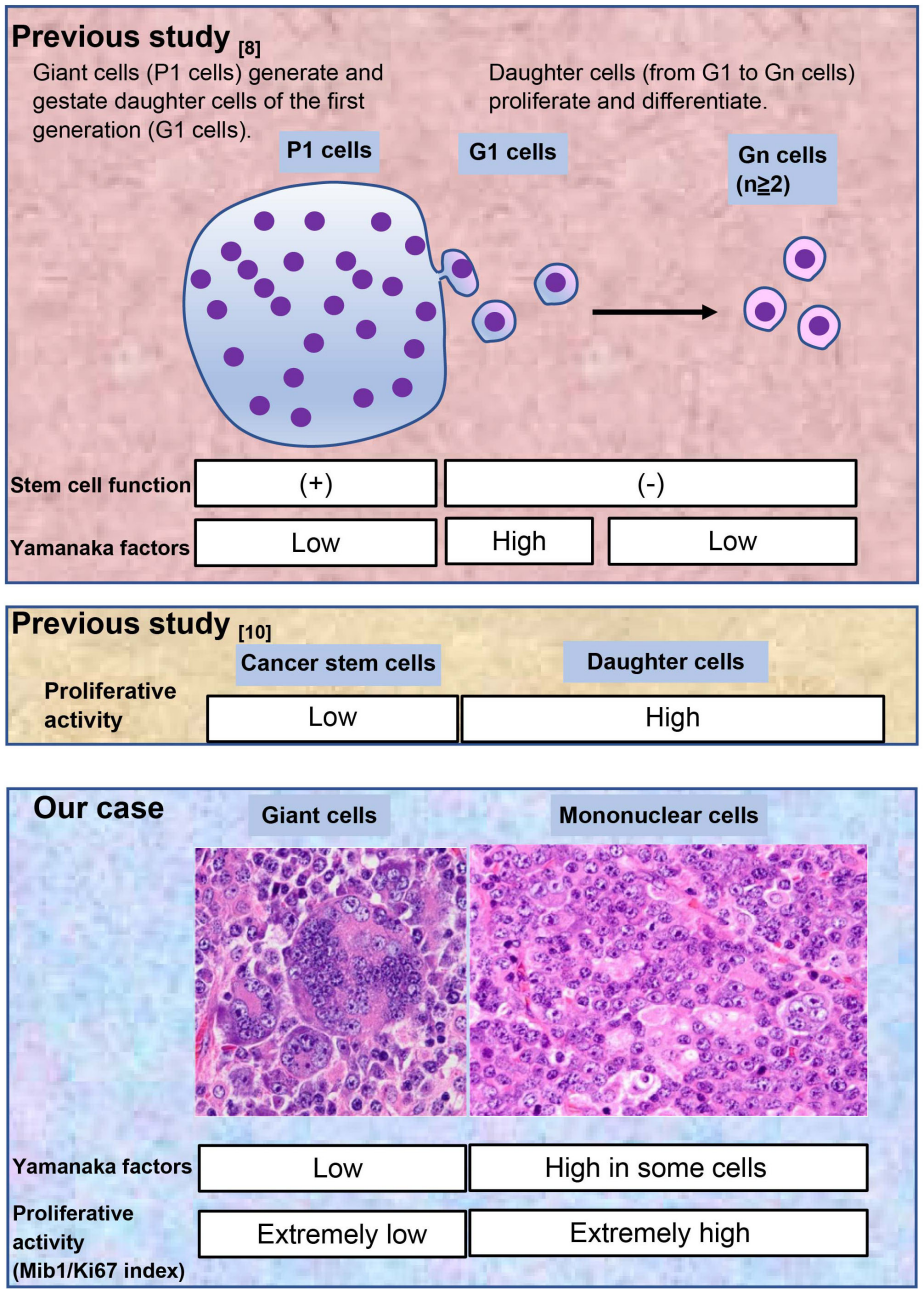
Comparison of our case with previous studies.

We have summarized these results in **Figure 3** and compared them to the results of previous studies (8, 10). This comparison is explained in detail in the discussion.

### Fluorescent *in situ* hybridization

Unstained sections (thickness, 4 µm) were “pretreated” using a Histology Fluorescent *in situ* hybridization (FISH) kit (GSP Laboratory, Kobe, Japan). Next, they were subjected to hybridization with BAC clone-derived probes for CCND1 and IGH, with a CKS1β dual-color probe set (Agilent) or with a c-Myc dual-color probe set (Abbott). The names of BAC clones used will be provided upon request. Hybridized slides were then stained with DAPI (4,6-diamidino-2-phenylindole, dihydrochloride) and examined using a fluorescence microscope BX51 (Olympus, Tokyo, Japan).

Split of CCND1 and/or IGH is a genetic marker for PPCM. However, neither was detected using FISH. Split of c-Myc is a genetic marker for PBL. However, it was not detected either. Moreover, amplification of CKS1β, a poor prognostic factor for PPCM (11), was also not detected.

## Discussion

In the present case, the patient was diagnosed with PBN, comprising predominantly proliferating mononuclear cells with admixed multinucleated giant cells, both of which were confirmed to be neoplastic. The analysis of stemness indicated that the multinucleated giant cells were compatible with cancer stem cells.

Diagnosis, in this case, was challenging owing to the difficulty in differentiating between PBL and PPCM. The EBER-ISH positivity suggested PBL; however, there were no indicators of immune suppression as the patient had no history of HIV infection or organ transplantation, and the tumor cells were negative for EBNA2, an indicator of immune suppression (12). Without immune suppression, a diagnosis of PBL was not strongly suggested. Furthermore, approximately 50% of PBLs harbor a rearrangement of c-Myc on chromosome 8 (13–15), while, in this case, there was no abnormality in chromosome 8. In addition, the expression of cyclin D1 suggested PPCM, but the cytogenetic analyses did not detect translocation or amplification of CCND1. Hence, the final pathological diagnosis was PBN.

Adult stem cells are associated with the capacity for self-renewal and multipotency. In neoplasms, putative cancer stem cells play these roles. Following chemo- and/or radiotherapy, multinucleated giant cells often emerge as cancer stem cells and are associated with tumor heterogeneity, therapy resistance, and metastasis (8, 9). Nonetheless, the hypothesis that neoplastic multinucleated giant cells serve as cancer stem cells has not been fully evaluated. We tested this hypothesis using the Yamanaka factors and Mib1/Ki67.

First, we evaluated the Yamanaka factors (OCT4, SOX2, KLF4, and c-Myc), which are implicated in cancer cell stemness (16, 17). The tumor consisted of mononuclear and multinucleated giant cells, and we assessed the stemness of both cell lineages. In a previous study on post-chemotherapy ovarian cancer, neoplastic multinucleated giant cells, designated as P1 cells, generated and gestated mononuclear daughter cells, designated as Gn cells (G1 cells were the first generation of Gn cells), and these cells were involved in drug resistance (8). Although P1 cells had stem cell functions, stem cell markers such as CD44 and Yamanaka factors were more strongly expressed in G1 than in P1 cells in that study. This unexpected phenomenon has not yet been fully explained. In concordance with the previous study (8), in our case, the stem cell markers OCT4, c-Myc, and KLF4 were more strongly expressed in the mononuclear than in the multinucleated giant cells. Moreover, SOX2 was negative in both cell types.

Next, we evaluated the proliferative activity. Stem cells generally grow slowly and have low proliferating activity (10). We used the Mib1/Ki67 index as an indicator of proliferative activity. In our case, mononuclear and multinucleated giant cells had a very high and very low Mib1/Ki67 index, respectively, which was interpreted as supportive evidence for the stemness of the multinucleated giant cells. Based on these results, we concluded that the multinucleated giant cells (P1 cells) were cancer stem cells.

Previous studies on neoplastic multinucleated giant cells have been conducted primarily after chemo- or radiation therapy. According to the study on ovarian cancer, multinucleated giant cells were rarely seen in untreated patients and markedly increased after chemotherapy (8).

In our case, there were several multinucleated giant cells even before chemotherapy, suggesting that the phenomenon of maternal multinucleated giant cells gestating mononuclear cells is not limited to the post-chemotherapy period. Moreover, we used formalin-fixed paraffin-embedded specimens for the analysis, ensuring high accessibility. The same analyses can be conducted on multiple cases at a low cost.

Considering the persisting scarcity of knowledge on cancer stem cells, we believe that the present case will add insight to the biology of cancer stem cells.

## Data availability statement

The original contributions presented in the study are included in the article/**Supplementary Material**. Further inquiries can be directed to the corresponding author.

## Ethics statement

The studies involving human participants were reviewed and approved by The Ethics Committee of Toho University. The patients/participants provided their written informed consent to participate in this study. Written informed consent was obtained from the individual(s) for the publication of any potentially identifiable images or data included in this article.

## Author contributions

NO-K and YS contributed to conception and design of the study. NO-K and TY conducted the immunohistochemical and molecular biological experiments. NO-K and YS were involved in the data analyses. NO-K wrote the first draft of the manuscript. YS wrote sections of the manuscript. NS and NH reviewed the manuscript. All authors contributed to manuscript revision, read, and approved the submitted version.

## Acknowledgments

The authors thank Dr. Kengo Takeuchi from the Cancer Institute, Japanese Foundation for Cancer Research, for his insightful suggestions. The authors are also grateful to Ms. Satoko Baba for preparing tests using fluorescent *in situ* hybridization. We would like to thank Editage (www.editage.com) for English language editing.

## Conflict of interest

The authors declare that the research was conducted in the absence of any commercial or financial relationships that could be construed as a potential conflict of interest.

## Publisher’s note

All claims expressed in this article are solely those of the authors and do not necessarily represent those of their affiliated organizations, or those of the publisher, the editors and the reviewers. Any product that may be evaluated in this article, or claim that may be made by its manufacturer, is not guaranteed or endorsed by the publisher.
