# Supplementary material for: Case report: Plasmablastic neoplasm with multinucleated giant cells—Analysis of stemness of the neoplastic multinucleated giant cells
Source: Front Oncol. 2022 Nov 29;12:1023785. doi: 10.3389/fonc.2022.1023785 (PMC9745170; doi:10.3389/fonc.2022.1023785)
Supplement: Supplementary file 1 [file DataSheet_1.pdf]

Supplementary Material

1 Supplementary Figures and Tables

1.1 Supplementary Figures

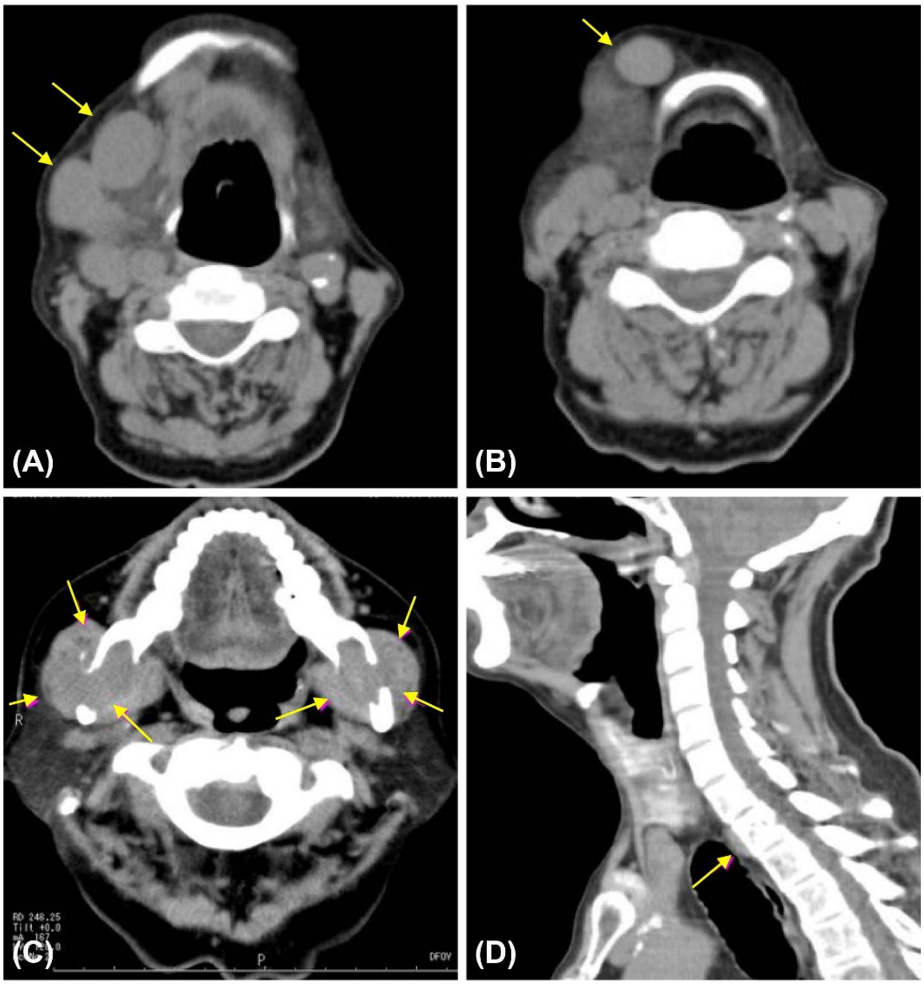

Supplementary Figure 1. Computed tomography images. Axial images showing enlarged lymph nodes (yellow arrows) in the cervical and head regions (A, B) and mandibular infiltration (C). Sagittal image showing infiltration of the Th1 vertebral body (D).

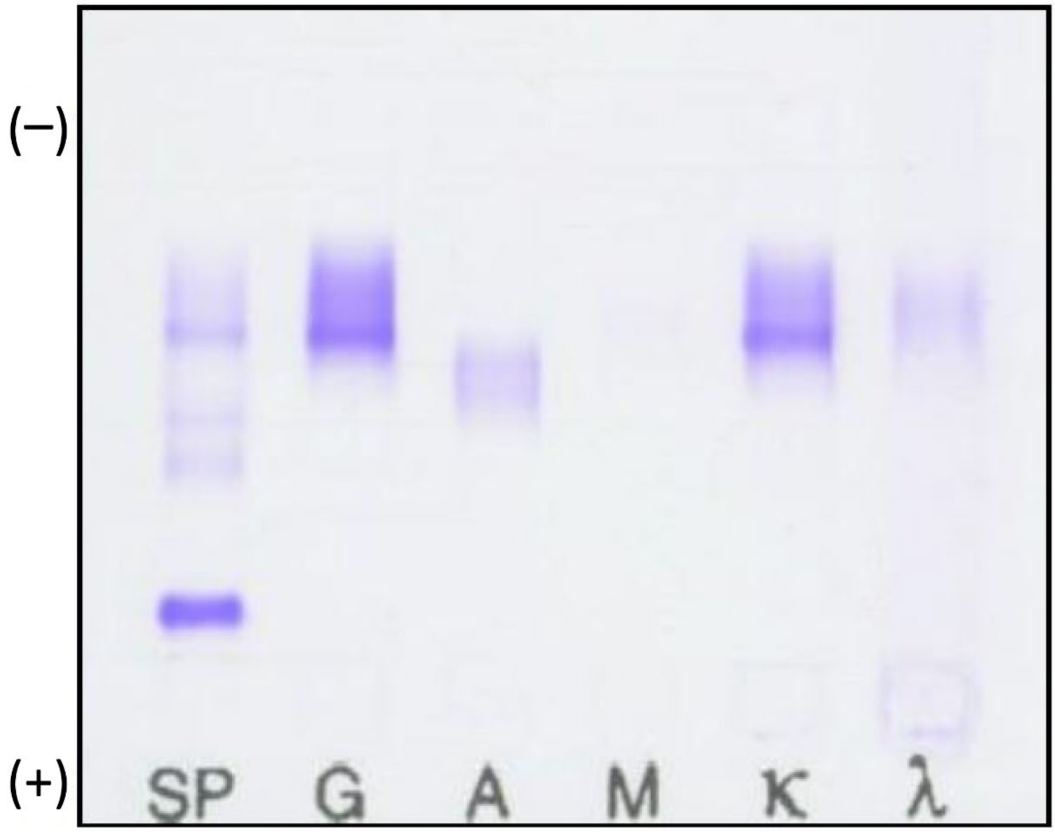

Supplementary Figure 2. Serum immune electrophoresis result

1.2 Supplementary Tables

Supplementary Table 1. Characteristics of PBL and PPCM

	**EBV infection**	**HIV infection**	**Cyclin D1**	**Bone lesions**	**Ig**	**Genetic abnormality**
**PBL**	+ or -	+ in approx.70%	-	rare	M or G	*MYC* rearrangement
**PPCM**	-	-	+ or -	+	G	Split of *CCND1* and/or *IgH*

EBV, Epstein-Barr virus; HIV, human immunodeficiency virus; Ig, immunoglobulin; PBL, plasmablastic lymphoma; PPCM, plasmablastic plasma cell myeloma

Supplementary Table 2. Results of laboratory tests

**Hematology**		**Chemistry**		**Serology**	
**WBC**	7,800/µL	CRP	1.94 mg/dL	IgG	2,193 mg/dL*
**Neu**	68.8%	TP	8.1 g/dL	IgA	306 mg/dL
**Stab**	1.0%	Alb	3.7 g/dL	IgM	65 mg/dL
**Seg**	63.5%	AST	14 U/L	β2MG (serum)	2.0 mg/dL
**Ly**	22.6%	ALT	10 U/L	HIV - 1/2	(-)
**Mo**	7.3%	LDH	166 U/L		
**Eos**	0.9%	ALP	360 U/L	**Coagulation**	
**Baso**	0.4%	T-Bil	1.1 mg/dL	PT	12.8 s
		BUN	12.6 mg/dL	PT-INR	1.03
**RBC**	401×10^4/µL	CRE	0.57 mg/dL	aPTT	28.4 s
**Hb**	13.1 g/dL	UA	4.0 mg/dL		
**Hct**	37.7%	Na	125 mEq/L		
**MCV**	94 fL	K	4.1 mEq/L		
**MCH**	32.9 pg	Cl	89 mEq/L		
**MCHC**	35.0%	Ca	9.1 mg/dL		
**PLT**	28.8×10^4/µL	IP	3.5 mg/dL		

**Ret**	28%	**Chromosome analysis**	
		46, XY, del (1) (p?), -13, -22, +2mar [7] /46, XY [13]	

*Reference value of IgG, 870−1, 700 mg/dL

WBC, white blood cells; Neu, neutrophils; Non-seg, non-segmented; Seg, segmented; Ly, lymphocytes; Mo, monocytes; Eos, eosinophils; Baso, basophils; RBC, red blood cells; Hb, hemoglobin; Hct, hematocrit; MCV, mean corpuscular volume; MCH, mean corpuscular hemoglobin; MCHC, mean corpuscular hemoglobin concentration; PLT, platelets; Ret, reticulocytes; CRP, C-reactive protein; TP, total protein; Alb, albumin; AST, aspartate aminotransferase; ALT, alanine aminotransferase; LDH, lactate dehydrogenase; ALP, alkaline phosphatase; T-Bil, total bilirubin; BUN, blood urea nitrogen; CRE, creatinine; UA, uric acid; Na, sodium; K, potassium; Cl, chloride; Ca, calcium; IP, inorganic phosphorus; Ig, immunoglobulin; β2MG, beta-2 microglobulin; HIV, human immunodeficiency virus; PT, prothrombin time; PT-INR, prothrombin time/international normalized ratio; aPTT, activated partial thromboplastin time.

Supplementary Table 3. Primary antibodies used in the analysis

Antibody	Supplier	Clone	Dilution
**CD138**	Agilent	MI15	RTU
**MUM-1**	Agilent	MUM1p	RTU
**CD20**	Agilent	L26	RTU
**CD79a**	Agilent	JCB117	RTU
**CD68**	Agilent	PG-M1	RTU
**Cyclin D1**	Agilent	EP12	1:50
**CD56**	Agilent	123C3	RTU
**IgG**	Agilent	Polyclonal	1:2
**κ**	Biocare Medical	L1C1	RTU
**λ**	Biocare Medical	N10/2	RTU
**CK (AE1/AE3)**	Agilent	AE1/AE3	RTU

CK (AE1/AE3), cytokeratin AE1/AE3; MUM-1, multiple myeloma oncogene-1; +, positive; -, negative; RTU, ready-to-use
